# A Gram Stain Hands-On Workshop Enhances First Year Medical Students' Technique Competency in Comprehension and Memorization

**DOI:** 10.1371/journal.pone.0163658

**Published:** 2016-10-06

**Authors:** Matthew S. Delfiner, Luis R. Martinez, Charles S. Pavia

**Affiliations:** Department of Biomedical Sciences, NYIT College of Osteopathic Medicine, New York Institute of Technology, Old Westbury, NY, United States of America; Rutgers University, UNITED STATES

## Abstract

**Background:**

Laboratory diagnostic tests have an essential role in patient care, and the increasing number of medical and health professions schools focusing on teaching laboratory medicine to pre-clinical students reflects this importance. However, data validating the pedagogical methods that best influence students’ comprehension and interpretation of diagnostic tests have not been well described. The Gram stain is a simple yet significant and frequently used diagnostic test in the clinical setting that helps classify bacteria into two major groups, Gram positive and negative, based on their cell wall structure.

**Methods and Findings:**

We used this technique to assess which educational strategies may improve students’ learning and competency in medical diagnostic techniques. Hence, in this randomized controlled study, we compared the effectiveness of several educational strategies (e.g. workshop, discussion, or lecture) in first year medical students’ competency in comprehension and interpretation of the Gram stain procedure. We demonstrated that a hands-on practical workshop significantly enhances students’ competency in memorization and overall comprehension of the technique. Interestingly, most students irrespective of their cohort showed difficulty in answering Gram stain-related analytical questions, suggesting that more emphasis should be allocated by the instructors to clearly explain the interpretation of the diagnostic test results to students in medical and health professional schools.

**Conclusion:**

This proof of principle study highlights the need of practical experiences on laboratory medical techniques during pre-clinical training to facilitate future medical doctors’ and healthcare professionals’ basic understanding and competency in diagnostic testing for better patient care.

## Introduction

Laboratory medicine, including diagnostic laboratory microbiology, pathology, hematology, and other testing, has been increasingly included in medical school pre-clinical curricula [1]. This addition has been made necessary by growing expenditures due to inappropriate or excessive diagnostic test ordering by physicians [1, 2]. Despite the inclusion of laboratory medicine to curricula in 52% of the United States allopathic medical schools [3], it has recently been reported that graduating medical students are not prepared to properly order and interpret laboratory diagnostic tests upon advancement in their medical training [4, 5]. To combat the lack of ability to properly interpret laboratory tests, various sample curricula have been proposed. These suggestions include early exposure to laboratory medicine, the use of small group discussions, and hands-on workshops [6, 7]. However, data demonstrating which educational method yields the greatest student comprehension is lacking [8].

This issue is common to most course subjects taught at medical schools throughout the United States, yet what makes laboratory medicine unique amongst the pre-clinical sciences is that this field has both cognitive and psychomotor components. The cognitive aspect is knowledge based, and it tracks advancement from simply remembering terms towards the ultimate goal of mastery [9]. The psychomotor component is hands-on, and it is used to develop a learner’s ability to become competent with specific skills [10]. Laboratory medicine fits within both of these domains, as the interpretation of diagnostic laboratory tests is cognitive, while the performance of the test is psychomotor. Because of this duality, the outcomes of training laboratory medicine can be explored in interesting ways to determine whether teaching the psychomotor component can actually aid the cognitive aspects.

The Gram stain is a simple yet commonly used laboratory technique that differentially classifies bacteria by the structure of its cell wall into two large groups: Gram positive and negative. This technique is a useful model to teach laboratory medicine, since it is a standard bacteriology method that pre-clinical students will learn and sometimes perform during their clinical rotations. Hence, we designed a randomly controlled study to determine whether hands-on Gram stain experience increases a student’s ability to interpret the results of this procedure. When comparing a cohort of students taking a hands-on laboratory workshop, a typical pre-clinical lecture, or a small focus group discussion, we hypothesized that the group of students who were immersed in a hands-on setting will achieve higher competence after taking an assessment quiz on the Gram stain procedure compared to the other educational methods. This proof of principle study suggests that practical training of laboratory medical techniques assists in the basic understanding of diagnostic testing by pre-clinical students and may facilitate the integration of the cognitive and psychomotor components for their learning.

## Materials and Methods

### Cohort groups

**Fig 1:**
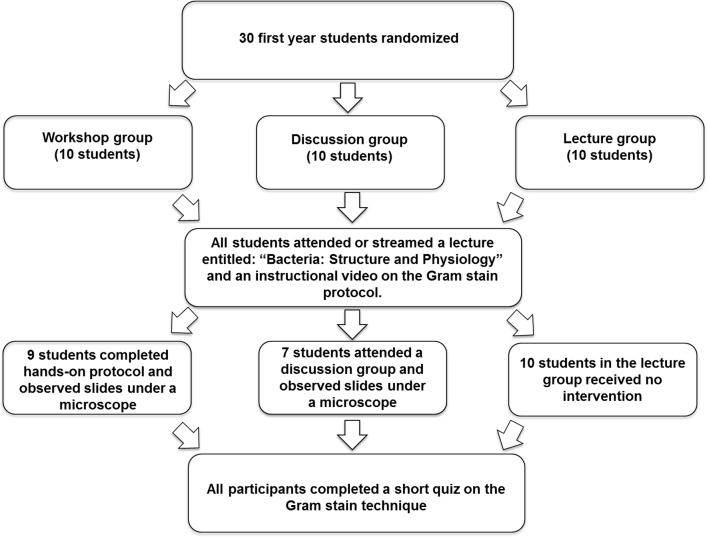
Study Design. Overview of the experimental plan comparing Gram stain procedure’s competency in medical students randomly assigned to a practical workshop, discussion, or lecture group.

Study volunteers were first year medical students at NYIT College of Osteopathic Medicine (NYITCOM). Thirty participants were initially enrolled in the study, with ten assigned to each of three separate groups. A total of four participants in both the workshop (*n* = 1) and discussion (*n* = 3) groups withdrew at various points in the study, resulting in a total of 26 subjects. Subjects were randomly assigned into three groups: a workshop group (average age: 23.9; 3 males and 6 females), a small discussion group (average age: 23.2; 1 male and 6 females), and a lecture group (average age: 24.4; 2 males and 8 females) (Fig 1). To minimize randomization bias, subjects who reported extensive microbiology education or professional experience were excluded from the study. Effective randomization minimized the likelihood of one group having a greater competency baseline than the other groups. All study participants attended or streamed a lecture entitled “Bacteria: Structure and Physiology” where they learned about the structure of bacterial cell walls and the basic principles of the Gram stain procedure. This lecture was taught in 50 minutes and is a component of the course “Foundations of Osteopathic Medicine” given to the first year NYITCOM medical students as part of their curriculum. In addition to the lecture, the workshop group participants also completed a 1 hour practical exercise where they performed the Gram stain procedure and observed their stained slides using an upright Olympus CH2 light microscope (Olympus, Tokyo, Japan) under the guidance of a microbiology professor. The discussion group met for 1 hour with a microbiology instructor, interpreted pre-stained slides observed under a microscope and discussed why the bacteria appeared as they did, as well as attending the lecture. Both the workshop and discussion groups were free to ask the facilitator questions about the technique protocol, interpretation of the results, and their significance. The lecture group did not have an intervention, and their lecture-only learning followed the same Gram stain education method of the typical NYITCOM student. All participants were given the opportunity to watch a 10 minutes online NYITCOM-produced instructional video that guided viewers through each step of the Gram stain protocol. Additionally, they were instructed for 30 minutes in biosafety practices (BSL-1 and 2) for teaching laboratories as described by the American Society for Microbiology (ASM) published guidelines (http://www.asm.org/index.php/education2/22-education/8308-new-version-available-for-comment-guidelines-for-best-biosafety-practices-in-teaching-laboratories). Each participant had previously taken an undergraduate microbiology course and been trained in the manipulation of BSL-1 microbes. For this study, BSL-2 guidelines were followed by the participants in the laboratory including standard laboratory practices (e.g. hand washing before and after the activity), personal protection requirements (e.g. safety glasses, gloves, coat, closed-toe shoes), and laboratory physical space requirements (e.g. biological safety cabinet (recommended), sink, eyewash station, lockable door to the room, etc.). The Institutional Review Board at NYITCOM approved this study (Protocol number: BHS 1138). All subjects provided verbal informed consent to participate in this study. Written consent was waived by the IRB because the study represented minimal risk for the participants.

### Gram stain

The Difco/BBL Gram stain kit (BD, Franklin Lakes, NJ) was used for performing the Gram stain procedure. The kit consists of 4 bottles of 1X solutions of crystal violet, Gram’s iodine, acetone-alcohol and safranin. The microscope slides, for use in this study, were prepared using fresh cultures of *Staphylococcus aureus* strain 6538 (a Gram-positive coccus; ATCC, Manassas, VA) and *Klebsiella pneumoniae* strain 13882 (a Gram-negative bacillus; ATCC) that had been maintained and grown for 24 h at 37°C on tryptic soy agar plates (BD). These BSL-2 microbes were selected because they are medically important pathogens and relevant to the participants. Smears of each bacterium were made onto separate slides and these were heat-fixed just prior to their use by the study participants. The kit reagents and the microscope slide preparations were examined before being used in the study to ensure their performance accuracy and to provide the expected staining outcomes. The Gram stain procedure was performed following the protocol described in a standard microbiology laboratory manual [11].

### Assessment

One week after the study interventions, the participants took an 11-question quiz (S1 Quiz), which assessed their understanding of Gram stain procedure, its significance, and interpretation. Ten questions were multiple-choice with each one worth one point. One closed-ended question listed each step of the Gram stain procedure and asked the participants to place each step in the correct order, and this question was worth 1 point (correct or incorrect) on the quiz. The highest score possible on the quiz was 11 points. Each question was categorized as memorization (Questions 1, 2, and 8), understanding (Questions 3–7), or analysis (Questions 9–11), and mean scores for each group within each category were calculated and analyzed.

### Statistical analysis

Statistical analyses were obtained utilizing GraphPad Prism 6.0 (GraphPad Software, La Jolla, CA) software. *P* values were calculated by analysis of variance (ANOVA) and were adjusted by use of the Bonferroni correction. *P* values of <0.05 were considered significant.

## Results

### Comparison of quiz mean scores

**Fig 2:**
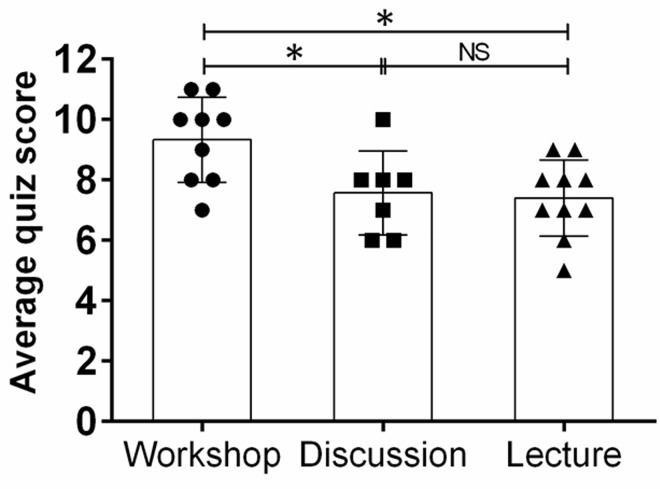
Mean quiz scores between cohorts. Students randomly assigned to a practical workshop group demonstrated higher average quiz score than those students in a discussion or lecture groups. Each symbol represents a subject, bars signify the average (workshop, *n* = 9; discussion, *n* = 7; lecture, *n* = 10) for each experimental condition, and error bars indicate standard deviations. *P* value significance (*, *P* < 0.01; NS, not significant) was calculated by ANOVA and adjusted by use of the Bonferroni correction.

To assess whether a practical workshop would enhance a cohort of first year medical students’ competency in comprehension and interpretation of Gram stain’s results, subjects were tested using a quiz, and mean score performances were compared to other cohorts of students taking a traditional lecture or small focus group discussion (Fig 2). On average, the workshop cohort demonstrated a significantly higher score (mean: 9.33; range: 7–11) than the discussion (mean: 7.57; range: 6–10) (*P* < 0.01) and lecture (mean: 7.4; range: 5–9) (*P* < 0.01) cohorts. There was no difference in score between the discussion and lecture groups. Notably, in the workshop group, there were 5 students with ≥ 10 correct questions whereas only 1 student in the discussion and none in the lecture groups had a similar number of correct questions.

### Comparison of correct questions per category

To determine the efficacy of each educational tool (e.g. workshop, discussion, or lecture) used in this study, the questions included in the quiz were categorized within three cognitive areas: memorization, understanding, and analysis. The percentage of correct questions per category for each cohort was calculated and compared to each other (Table 1). Students in the workshop group evinced a higher percentage of correct questions in each category compared to the other groups. In the memorization category, we observed that students in the workshop group (88.89%) had a significantly higher percentage of correct questions relative to students in the lecture group (50%) (*P*<0.05). Although there was a trend when the percentage of correct questions in the workshop group (88.89%) was compared to the discussion group (66.67%), it was not statistically significant. There was no statistical difference in the understanding and analytical categories when the cohorts were compared. The mean percentages of correct questions in the understanding category for students assigned to the workshop, discussion, and lecture groups were 91.11%, 74.29%, and 86%, respectively. Likewise, the mean percentages of correct questions in the analysis questions were 70.37%, 61.90%, and 53.33% for students assigned to the workshop, discussion, and lecture cohorts, respectively.

**Table 1:** Question category per cohort. Percentages of the students’ correct questions per category after taking a Gram stain procedure quiz.

Category of questions	Workshop (%)	Discussion (%)	Lecture (%)
Memorization	88.89*	66.67	50
Understanding	91.11	74.29	86
Analytical	70.37	61.90	53.33

*, *P*<0.05; indicates significance compared to lecture group.

## Discussion

A major challenge in medicine is making the leap from the medical training years to patient encounters in the clinical setting. The ability of recently graduated medical doctors to order diagnostic tests and interpret their results has been scrutinized, suggesting that medical schools do not properly prepare students for this critical task. Despite the efforts implemented by medical schools in including laboratory medicine in their curriculum, there is a dearth of information on the teaching methods that yield the best students’ learning outcomes. Therefore and as a proof of principle, we asked whether practical experience performing a laboratory technique such as the Gram stain increases competence in the comprehension and interpretation of the procedure by first year medical students.

Our findings demonstrated that hands-on experience considerably enhances students’ laboratory technique competency in comprehension and memorization compared to those exposed to other learning methods. It is conceivable the workshop cohort’s high scores were heavily influenced by the amount of time students spent in close proximity with an instructor who explained the stain procedure step by step. In this regard, five out of nine students attending the workshop obtained ≥10 correct questions. However, the discussion cohort group spent an equal number of classroom hours with the same instructor and only 1 student had a score of 10. In contrast, the students in the lecture group did not spend additional time with the instructor, and this cohort did not have any students with a score >9. These results reveal that the practical experience of the workshop, rather than the additional exposure to the Gram stain didactic material, was important in scoring high on the assessment quiz compared to the other groups. These outcomes are consistent with other studies that suggest the importance of active learning and student-centered pedagogy, especially in the sciences [12, 13]. While previous education-based research has identified peer discussion as a factor that improves academic competence [14], it is clear that in our study a practical experience provides a unique and advantageous learning aspect in students’ laboratory technique competency, given that there was no difference in the mean quiz score between the discussion and lecture groups and considering that the participants have comparable educational preparation. Previous studies in pedagogical methods have demonstrated that students exposed to hands-on practical experiences have a deeper understanding of the concepts taught in the classroom than the students who only have lecture-based lessons [15]. Our findings support the idea that hands-on learning is more likely to engage students, but also that it can actually boost comprehension in diagnostic techniques.

To better understand the impact of the different teaching methods utilized in this study, we clustered the questions of the assessment in various cognitive categories including memorization, understanding, and analysis. We found that the workshop cohort of students had better percentages of correct questions in every category than students assigned to the discussion or lecture groups. Particularly, we observed that hands-on experience provided by the workshop significantly enhanced students memorization. Interestingly, students having only a lecture showed a better understanding of the procedure than the discussion group. Furthermore, the majority of the students regardless of their cohort demonstrated lower percentage of correct answers to the analysis questions, suggesting that perhaps more efforts should be allocated by instructors to extensively explain the interpretation of the diagnostic test results to students. Likewise, the fact that only half of the students in the lecture group answered correctly the analysis questions indicates that involvement of students in active learning and practical experiences are necessary for mastering diagnostic techniques. Nonetheless, it is important to describe several limitations in implementing hands-on training sessions into medical school curricula. For example, multiple instructors are usually needed to keep low student to teacher ratio, particularly in medium to large size medical schools to maximize student learning. In this regard, complications such as scheduling difficulties and additional preparation time may arise due to other instructors’ professional responsibilities, whether they are researchers, administrators, or teachers of several courses [3]. This is a major challenge in medical education given that some teachers may opt-out of participating in project-based or hands-on learning because of the extra time and preparation required to set those lessons up.

Despite the Gram stain being an important medical laboratory technique, our study is limited and one cannot extrapolate the results presented here to the broad variety of diagnostic tests used in the clinical setting. Similarly, it may be difficult to extrapolate our findings to a greater population, since the act of volunteering for this study by the participants demonstrates a possible eagerness to learn that other students may not possess. Nevertheless, this report establishes a proof of principle to carry out similar studies in the future in order to understand the importance of practical interventions during medical doctors and other health professionals training, especially in diagnostic testing and analysis. Students’ early exposure to diagnostic techniques preferably as undergraduates or at the initial steps of their medical education may enhance their critical thinking, diagnostic skills, and clinical decision-making.

## Supporting Information

S1 Quiz Gram stain procedure quiz. An 11 question post-workshop quiz was given to the subjects 1 week after the study interventions, to assess their understanding of Gram stain procedure, its significance, and interpretation.
(DOCX)
