# Supplementary material for: A Gram Stain Hands-On Workshop Enhances First Year Medical Students' Technique Competency in Comprehension and Memorization
Source: PLoS One. 2016 Oct 6;11(10):e0163658. doi: 10.1371/journal.pone.0163658 (PMC5053596; doi:10.1371/journal.pone.0163658)
Supplement: S1 Quiz — An 11 question post-workshop quiz was given to the subjects 1 week after the study interventions, to assess their understanding of Gram stain procedure, its significance, and interpretation. (DOCX) [file pone.0163658.s001.docx]

COVER PAGE

Student Identifier:

For this study, to which group were you assigned?

1. Hands-on workshop (staining slides)
2. Small group workshop (observing slides but not creating them)
3. Control (Lecture only)

Demographic Questions (OPTIONAL)

1. What is your age? _______________
2. What is your gender? _______________
3. What is your race? _______________

-------------------------------------------------------------------------------------------------------

Instructions:

1. Please answer the questions that follow on the next few pages.
2. Put your correct ANSWER choice in the space provided for each question.
3. The Gram stain procedure is designed to stain which of the following?
4. Only bacteria that have a cell wall thick enough to retain the stain
5. Only bacteria that are lacking spores or peritrichous flagella
6. Only bacteria that can be isolated from a patient sample or from the environment
7. All types of bacteria, and certain types of fungi and protozoans
8. All types of bacteria, and certain types of fungi and viruses ANSWER____**A**____
9. The different ingredients for a Gram stain are reproduced here. Please number them in the correct order:

_**3**_Ethanol (Decolorizer) _**1**_Crystal Violet _**5**__Safranin _**2**_Iodine _**4**_Water

1. These bacteria can most accurately be described as:


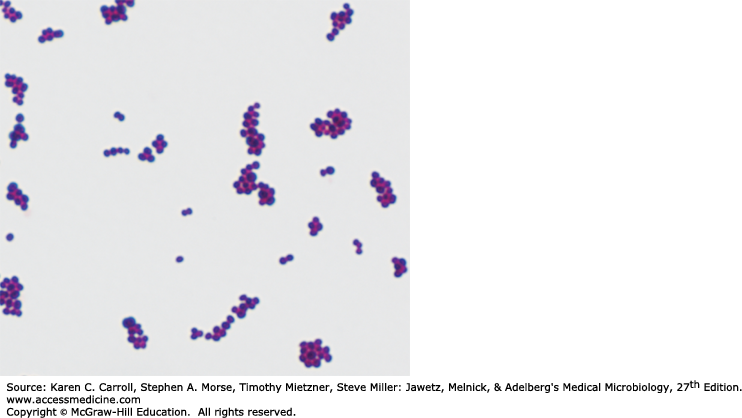


(Image source: UCSF Microbiology Image Archive <http://labmed.ucsf.edu/labmanual/clinlab/micro_images/>)

The image here showed purple-colored cocci arranged in clumps.

1. Gram positive bacilli
2. Gram positive cocci
3. Gram negative bacilli
4. Gram negative cocci

ANSWER____**B**_____

1. These bacteria can most accurately be described as:


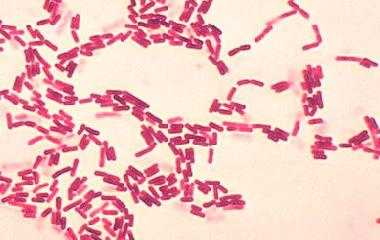


(Image source: Todar’s Online Textbook of Bacteriology; <http://textbookofbacteriology.net/Bacillus.html>)

The image here showed red-colored bacilli.

1. Gram positive bacilli
2. Gram positive cocci
3. Gram negative bacilli
4. Gram negative cocci ANSWER____**C**____
5. These bacteria can most accurately be described as:


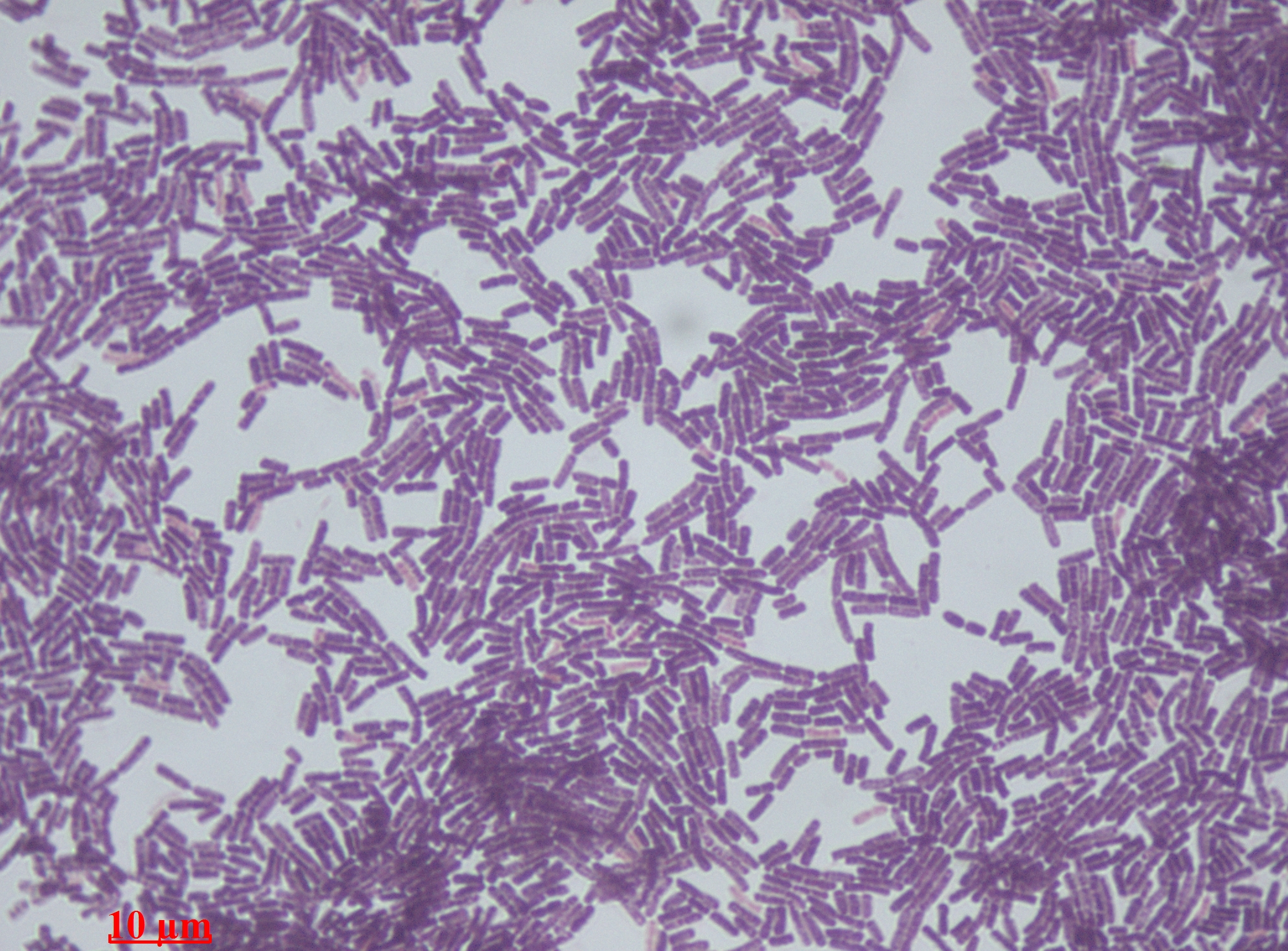


(Image source: Wikipedia; <https://en.wikipedia.org/wiki/Bacillus>)

The image here showed purple-colored bacilli.

1. Gram positive bacilli
2. Gram positive cocci
3. Gram negative bacilli
4. Gram negative cocci ANSWER____**A**____
5. What property of the bacteria, shown below, allows them to appear this color after performing the Gram stain?


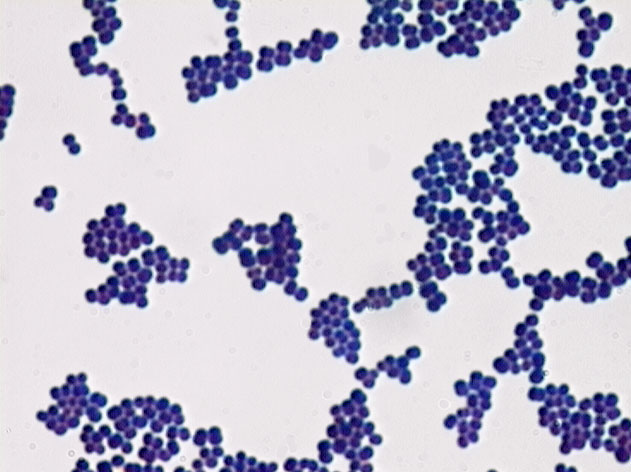


(Image source: Microbiology 101: the Gram stain; <http://www.lcscss.com/microbiology-101-the-gram-stain/>)

The image here showed purple-colored cocci.

1. The lipopolysaccharide is able to interact tightly with purple dye.
2. The peptidoglycan is able to interact tightly with the purple dye.
3. The phospholipids are able to interact tightly with the purple dye.
4. The teichoic acid is able to interact tightly with the purple dye.
5. The capsule is able to interact tightly with the purple dye. ANSWER____**B**____
6. After alcohol is added as decolorizer when performing the Gram stain, and if the procedure were stopped at that step, Gram (–) cells would appear:
7. Blue
8. Red
9. Purple
10. Colorless
11. Violet ANSWER____**A**____
12. After staining a prepared sample using the Gram stain, the sample is typically visualized using the 100x objective of a light microscope with the aid of immersion oil. What is the purpose of the oil in this regard?
13. To enable the stained sample to maintain its shape and appearance.
14. To allow for enough light to pass through from the condenser.
15. To enhance the resolution of the image when viewing the sample.
16. To keep the stained sample from detaching itself from the slide.
17. To prevent the objective lens from crushing the stained sample. ANSWER___**C**____
18. Craig performed a side-by-side Gram stain of a known Gram (+) and Gram (-) organism. Unfortunately, he forgot to do the iodine step. When he observed his completed slide under oil immersion, what do you think he saw?
19. Both Gram (+) and (-) organisms appear purple.
20. Both Gram (+) and (-) organisms appear red.
21. Both Gram (+) and (-) organisms appear colorless.
22. Both Gram (+) and (-) organisms are unidentifiable.
23. No organisms – all of them had washed off the slide. ANSWER____**B**____
24. As an internal medicine resident, you have a strong suspicion that a patient has an infection caused by a Gram negative organism. When you send a sample for a Gram stain, the results come back as Gram positive. Confused, you walk to the hospital’s microbiology lab to view the slide yourself and you notice the Gram stain shows what appears to be a purple organism. The clinical picture still points towards a Gram negative organism, so you repeat the stain yourself. You then view the new slide and see red cells. What is the most likely mistake that the original lab technician made when preparing the first slide?

A) He used too much safranin on the slide.

B) He did not use crystal violet for a long enough amount of time.

C) He forgot to decolorize the slide at the right time.

D) He did not wash the decolorizer off the slide with water.

E) He used an acid-alcohol solution as the decolorizing agent. ANSWER____**C**____

1. In your private practice, you see two patients in a row, both with possible infections. You take samples from each with the intent of sending them to a commercial laboratory for staining and culture. Unfortunately, the lab is closed. You remember you still have your Gram stain materials from your time as a medical student, and you perform the procedure on your own (see figure below for results). How might treatment differ between the two patients?


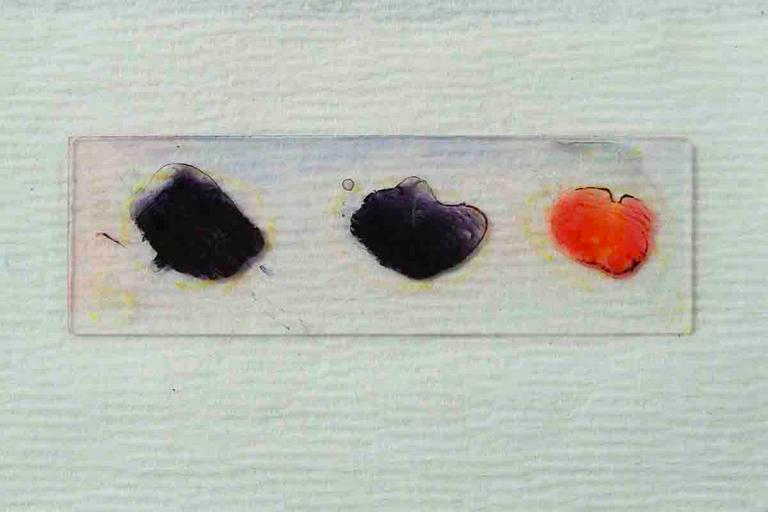


Control (i) (ii)

Purple Purple Red

(Image source: Gram stain for identifying bacteria; <http://www.scienceprofonline.com/microbiology/gram-stain-test-for-gram-positive-gram-negative-bacteria-id-2.html>)

1. Patient (i) can be treated with an antibiotic that targets peptidoglycan, while (ii) cannot.
2. Patient (ii) can be treated with an antibiotic that targets peptidoglycan, while (i) cannot.
3. Patient (i) can be treated with an antibiotic that targets safranin, while (ii) cannot.
4. Patient (ii) can be treated with an antibiotic that targets safranin, while (i) cannot.
5. Patient (i) can be treated with an antibiotic that targets the cell membrane while (ii) cannot.
6. Patient (ii) can be treated with an antibiotic that targets the cell membrane while (i) cannot.

ANSWER____**A**____
